# Characterization of uncertainty in the classification of multivariate assays: application to PAM50 centroid-based genomic predictors for breast cancer treatment plans

**DOI:** 10.1186/2043-9113-1-37

**Published:** 2011-12-23

**Authors:** Mark TW Ebbert, Roy RL Bastien, Kenneth M Boucher, Miguel Martín, Eva Carrasco, Rosalía Caballero, Inge J Stijleman, Philip S Bernard, Julio C Facelli

**Affiliations:** 1 Department of Biomedical Informatics, University of Utah, Salt Lake City, UT, USA; 2 ARUP Institute for Clinical and Experimental Pathology, Salt Lake City, UT, USA; 3 Department of Oncological Sciences, University of Utah, Salt Lake City, UT, USA; 4 Department of Medical Oncology, Hospital General Universitario Gregorio Marañón, Madrid, Spain; 5 Spanish Breast Cancer Research Group, GEICAM, Madrid, Spain; 6 Department of Pathology, Huntsman Cancer Institute/University of Utah, Salt Lake City, UT, USA; 7 Center for High Performance Computing, University of Utah, Salt Lake City, UT, USA

**Keywords:** Multivariate Assays, PAM50, Monte Carlo Simulations, Breast Cancer

## Abstract

**Background:**

Multivariate assays (MVAs) for assisting clinical decisions are becoming commonly available, but due to complexity, are often considered a high-risk approach. A key concern is that uncertainty on the assay's final results is not well understood. This study focuses on developing a process to characterize error introduced in the MVA's results from the intrinsic error in the laboratory process: sample preparation and measurement of the contributing factors, such as gene expression.

**Methods:**

Using the PAM50 Breast Cancer Intrinsic Classifier, we show how to characterize error within an MVA, and how these errors may affect results reported to clinicians. First we estimated the error distribution for measured factors within the PAM50 assay by performing repeated measures on four archetypal samples representative of the major breast cancer tumor subtypes. Then, using the error distributions and the original archetypal sample data, we used Monte Carlo simulations to generate a sufficient number of simulated samples. The effect of these errors on the PAM50 tumor subtype classification was estimated by measuring subtype reproducibility after classifying all simulated samples. Subtype reproducibility was measured as the percentage of simulated samples classified identically to the parent sample. The simulation was thereafter repeated on a large, independent data set of samples from the GEICAM 9906 clinical trial. Simulated samples from the GEICAM sample set were used to explore a more realistic scenario where, unlike archetypal samples, many samples are not easily classified.

**Results:**

All simulated samples derived from the archetypal samples were classified identically to the parent sample. Subtypes for simulated samples from the GEICAM set were also highly reproducible, but there were a non-negligible number of samples that exhibit significant variability in their classification.

**Conclusions:**

We have developed a general methodology to estimate the effects of intrinsic errors within MVAs. We have applied the method to the PAM50 assay, showing that the PAM50 results are resilient to intrinsic errors within the assay, but also finding that in non-archetypal samples, experimental errors can lead to quite different classification of a tumor. Finally we propose a way to provide the uncertainty information in a usable way for clinicians.

## Background

Multivariate assays (MVAs) using gene expression as their contributing factors are becoming commonly used in assisting treatment decisions in medicine, especially in oncology. Examples of MVAs available for planning breast cancer treatment include the 55-gene subtype classifier (PAM50 Breast Cancer Intrinsic Classifier) [1], the 21-gene prognosis assay (*Onco*type DX^®^) [2], the 14-gene prognosis assay (BreastOncPx™) [3] and the 70-gene prognosis assay (MammaPrint^®^) [4]. Though physicians may rely on these MVAs for planning treatment, little is known about the effects on the results of an assay due to the intrinsic error in the laboratory process and measuring its contributing factors - in this case, all steps required for preparing a sample (post RNA extraction), preparing the assay, and the instrumental errors for measuring gene expression. While we expect that classification of samples in proximity to one of the centroids defining the tumor classes, which we will call archetypal samples here, will be very stable with respect to experimental errors in the gene expression measurements, what happens to the samples not in proximity to one centroid is unknown. For example, if a sample lies in a "gray" area where the intrinsic errors in the gene expression measurements may result in a change of its classification each time the sample is run, the results reported to the attending physician may be misleading because he/she is getting the results from only one measurement and no information about the probability for sample misclassification. Given the serious consequences due to ambiguous results in clinical classifications, methods to measure the effects of an MVA's intrinsic errors need to be established and communicated to attending physicians.

The PAM50 assay measures the expression level of 55 genes (50 classifier genes and 5 housekeepers) creating a "signature" that is compared, using Spearman's Rho as a distance measure, to each of five centroids [1,5] representing the Luminal A, Luminal B, HER2-enriched and Basal-like subtypes [1,6,7], as well as Normal-like tissue.

The complexity of the PAM50 assay demonstrates the challenge of identifying and understanding error sources from the moment a sample is received by the reference laboratory until a clinical report is generated. Error sources to be considered include heterogeneity and sample preparation, as well as technical variability, which may be separated into error from measuring devices as well as intrinsic and explicit classification uncertainties. While the overall purpose of this project is to develop a model for estimating the effect of all error sources on final MVA results, in this paper we focus on how to estimate the effect that intrinsic gene expression measurement errors, including those associated with sample and assay preparation, have on the classification of tumors.

## Methods

A comprehensive experimental study to estimate the effect that the intrinsic gene expression measurement errors have on the classification of tumors and gene-score classifications requires, in principle, repeated testing of a significant number of samples from each subtype and thorough analysis of the misclassifications observed. Such a comprehensive approach is unfeasible in terms of cost and sample availability. Here we have adopted a hybrid approach in which we perform repeated experimental measurements on one sample from each subtype (i.e. Luminal A, Luminal B, HER2-enriched and Basal-like) to determine the experimental variability of the measured gene expression for each of the 50 genes included in the PAM50 assay. Using this experimental information we proceed to generate a Gaussian error distribution that can be used to generate multiple data sets by way of Monte Carlo simulations. These simulations impose random errors, given by the Gaussian distribution, on the set of experimental measured samples. Monte Carlo simulations are well suited for this hybrid analysis because there is extensive literature for using this approach to estimate errors in high-dimensional problems, such as fluids and thermodynamics [8-10], where conventional analytics are not feasible [11,12]. The simulated data sets are then classified by the standard PAM50 algorithm, and the misclassifications encountered in the synthetic data sets are used as a proxy for the PAM50's misclassification rate based on the assay's intrinsic error.

Specifically, in this study we followed five major steps: (1) collect and prepare four archetypal samples representative of each cancer subtype; (2) characterize the intrinsic error for each gene's expression values in the assay by making twelve measurements for each gene's expression on each archetypal sample and determine the distribution type that best models the experimental errors; (3) using Monte Carlo simulations generate a sufficient number of simulated test samples, based on a defined confidence interval width, by imposing the errors generated using the distribution from (2) onto the archetypal samples; (4) determine the effect of the variability imposed in the simulated samples on their classification; and (5) repeat steps (3) and (4) on an independent set of samples from the GEICAM 9906 clinical trial (GEICAM) [13].

### Archetypal Sample Collection and Preparation

In order to characterize the error in gene expression measurements, four archetypal samples representative of each cancer subtype with sufficient genetic material were constructed - since most single samples do not have enough genetic material to be tested more than twice. Cell lines representative of Basal-like (ME16C) and Luminal B (MCF7) subtypes were grown in the Reagent Lab at ARUP Laboratories. Luminal A and HER2-enriched subtypes were not readily available as a cell lines. As such, 20 patient tumor samples previously identified as archetypal Luminal A (10 samples) and HER2-enriched (10 samples), based on PAM50 gene scores and classification, were collected under IRB approved protocols at the University of Utah to be combined and treated as single tumor samples.

RNA was extracted from tumor-enriched areas of formalin-fixed, paraffin-embedded (FFPE) tissue blocks, containing more than 70% tumor cells, as determined during review by a board-certified pathologist. Samples were deparaffinized using Citrus Clearing Solvent (Richard-Allen Scientific, Kalamazoo, MI, http://www.thermofisher.com) followed by dehydration in absolute ethanol. RNA extraction was completed on a Biomek NX Laboratory Automation Workstation (Beckman Coulter, Beverly, MA, http://www.beckmancoulter.com) using the AgenCourt FormaPure Kit (Beckman Coulter, Beverly, MA, http://www.beckmancoulter.com) according to the manufacturer's instructions and including a DNase I step. RNA quantification was done on a Paradigm Detection Platform (Beckman Coulter, Beverly, MA, http://www.beckmancoulter.com) using the Quant-iT RiboGreen Assay Kit (Invitrogen, Carlsbad, CA, http://www.invitrogen.com). cDNA synthesis was performed on the Biomek FX Laboratory Automation Workstation (Beckman Coulter, Beverly, MA, http://www.beckmancoulter.com) using 600 ng of RNA, uracil containing dNTPs (Invitrogen, Carlsbad, CA, http://www.invitrogen.com), random primers (Invitrogen, Carlsbad, CA, http://www.invitrogen.com), gene-specific, downstream PCR primers (Idaho Technology, Salt Lake City, UT, http://www.idahotech.com ), and SuperScript III Reverse Transcriptase (Invitrogen, Carlsbad, CA, http://www.invitrogen.com ).

Each 5 μL reaction contained 1X LightCycler 480 SYBR Green I Master Mix (Roche Applied Sciences, Indianapolis, IN, http://www.roche-applied-science.com) and 1.67 ng cDNA were added to the experimental sample wells. Sample cDNA was incubated with LightCycler Uracil-DNA Glycosylase (Roche Applied Sciences, Indianapolis, IN, http://www.roche-applied-science.com) at 40°C for 10 min and inactivated at 95°C for 10 min prior to performing Real-time, quantitative PCR (RT-qPCR). RT-qPCR was performed on the LightCycler (LC) 480 (Roche Applied Sciences, Indianapolis, IN, http://www.roche-applied-science.com) as follows: 45 cycles at 95°C for 4 sec, 58°C for 6 sec and 72°C for 6 sec. To assure target specificity, RT-qPCR was followed by a melting curve analysis: 95°C for 15 sec, 65°C for 1 min followed by raising the temperature to 99°C while taking 10 fluorescence acquisitions/°C. We then classified the RT-qPCR data from each run. One run from the Luminal A sample failed quality control and was not included in further analysis.

### Error Characterization

**Figure 1:**
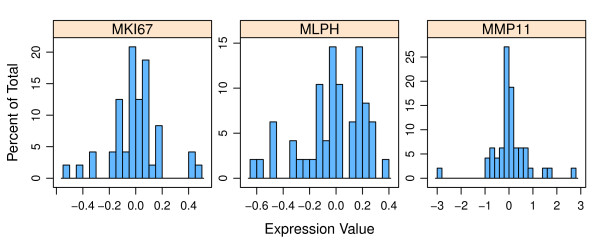
**Representative error distributions of the expression value used to fit the Gaussian distribution used in this work**. A complete set of the error distributions is given in Additional file 1.

In order to estimate the intrinsic experimental error in gene expression measurements within our laboratory, we performed twelve measurements for each gene on each archetypal sample. Each of the four archetypal samples was separated into, and treated as, twelve individual samples, and measured by RT-qPCR on the Roche LightCycler (LC) 480 (Roche Applied Sciences, Indianapolis, IN, http://www.roche-applied-science.com). The error distribution function type could not be estimated using only the twelve measurements for each gene within a given sample subtype, therefore to determine the error distribution function type for each gene, all four sample subtypes were median-centered by gene and combined, giving forty-seven data points per gene, since one of the archetypal Luminal A samples failed quality control. As depicted in Figure 1, the resultant error distributions for each gene can be reasonably approximated by Gaussian distributions. Therefore two hundred Gaussian distributions were generated, one per gene within each archetypal sample, using the mean and standard deviation of the twelve data points within the given gene and archetypal sample. Note that only the twelve data points available for each gene were used to determine the mean and standard deviation because the mean and standard deviation must be specific to the gene and subtype, whereas all forty-seven data points per gene were necessary to form a recognizable distribution. Gaussian distributions were generated using the "rnorm" function within *The R Statistical Package *(*R*) [14].

### Sample Size Justification for Archetypal Sample Monte Carlo Simulation

Before performing the Monte Carlo simulations an analysis to justify sample size was performed to ensure sufficient confidence for the analysis when using our target of 100,000 simulated samples. We calculated the 95% confidence interval width around the percentage of correct classifications using 100,000 simulated samples for each archetypal sample. For a dichotomous variable (i.e. misclassified or not), a confidence interval width (W) can be calculated (Eq. 1) given an alpha

(1) $$W=\pm Z_{\alpha }\sqrt{\frac{\theta \left(1-\theta \right)}{\pi }}$$

level (Z_0.05 _= 1.96), expected proportion of misclassifications (*θ *= 0.02) and the sample size, or in this case, the number of simulations (n = 100,000). The calculated confidence interval width is ± 0.00087 for each simulation of 100,000 samples, which is an acceptable value.

### Monte Carlo Simulation Using Archetypal Samples

Monte Carlo simulations were performed using the mean (μ) value and standard deviation (σ) for the expression of each gene within the archetypal samples, as described above. This procedure created a total of two hundred independent distributions, i.e. fifty Gaussian distributions (one for each gene) for each of the four archetypal samples. For example, the mean expression value (μ) for ACTR3B from the twelve Luminal B values was 1.94 and the corresponding standard deviation (σ) was 0.085. Therefore the ACTR3B expression value for each of the 100,000 simulated Luminal B samples was randomly selected from a Gaussian distribution centered on a mean (μ) of 1.94 and with a standard deviation (σ) of 0.085. Randomly selecting a value from the Gaussian distribution, as described, does not assume gene expression values are independent of one another, rather the method assumes that the measurement error for each gene is independent. Specifically, since each Gaussian distribution is centered on the gene's mean expression value for the given sample, any genes within the sample that are generally upregulated will all be generally upregulated in the simulated samples, while allowing the error to deviate independently. The 100,000 simulated samples for each archetypal sample were classified using the standard PAM50 process. The effect of intrinsic gene expression measurement error on the tumor classification was assessed by determining the percentage of simulated samples that were classified identically to the original sample. This value provides an estimate of the reproducibility of the results for archetypal samples.

### Evaluation Using the GEICAM Larger and Independent Test Set

Testing archetypal samples is valuable for determining how the PAM50 assay will perform under ideal circumstances, but these results may not be informative when the samples are not as well characterized as the archetypal samples. Thus, the method described above for the archetypal samples was adapted and applied to the larger and more diverse set of independent samples from the GEICAM 9906 clinical trial. A total of 911 breast tumors collected by the GEICAM group for the GEICAM 9906 clinical trial were run and classified by the PAM50. Tumor samples were prepared following the same methods described above for the archetypal samples and those with insufficient tumor content to be classified were excluded from further analyses.

### Monte Carlo Simulation Using GEICAM Samples

**Figure 2:**
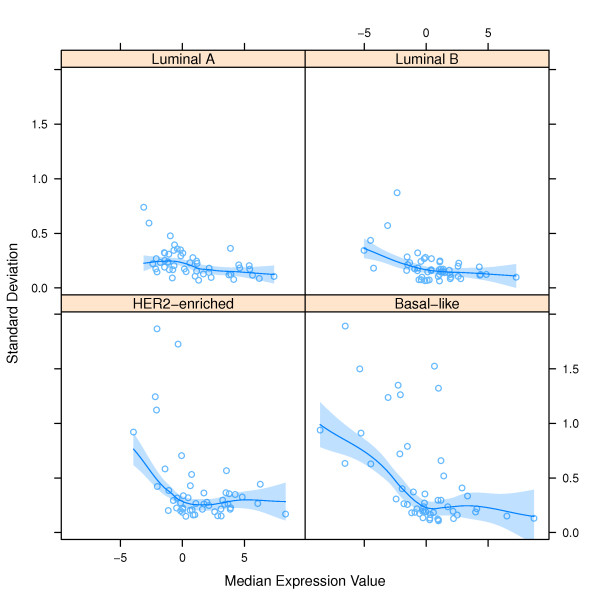
**Standard deviation as function of expression values and tumor subtype and loess model predicting standard deviation based on subtype and expression**. See text for description of the model development.

As depicted in Figure 2, the data from the multiple measurements in the archetypal samples show that standard deviation depends on the relative average gene expression value and on the sample subtype. To understand the sample subtype dependence one should understand that the expression values of the genes defining the expression pattern for each of the cancer subtype are quite different, e.g. the Luminal A subtype expresses all 50 genes at a level that is more easily quantified, producing a lower standard deviation; however, the HER2-enriched subtype expresses some genes at lower levels such that they are less easily quantified, producing a higher standard deviation. Therefore as depicted in Figure 2, the relative errors in the gene expression measurements in a Luminal A sample are smaller than those in a HER2-enriched sample. Accordingly our methods to produce simulated samples have to be modified to take into account these dependencies when applied to a set of non-archetypal samples. Using locally weighted scatter plot smoothing (loess), based on the PAM50's characterized error functions depicted in Figure 2, we developed error distributions that can be used to impose the error on individual test samples dynamically. The loess model was fit using the *R *function "loess" (span = 0.75, degree = 1, surface = "direct" and family = "symmetric") and graphed using "panel.smoother" from the *R lattice *graphics package. A 95% confidence interval for the fitted line was also calculated to test "best-case" (lower limit of the 95% confidence interval), "worst-case" (upper limit of the 95% confidence interval) and "average-case" (the fitted line) scenarios for subtype reproducibility. The "worst-case" scenario is considered such because it uses the highest estimated standard deviations, or error. Once the loess models were developed, we used these models to predict the standard deviation (σ) to generate Gaussian distributions for the Monte Carlo simulation. Specifically, given the test sample's original subtype and the expression value for a given gene, we used the loess model to predict the standard deviation (σ), or error, to be used in the Gaussian distribution for said gene and sample. The expression value was used as the mean of the Gaussian distribution. We repeated this process for all genes within each sample.

The model described above was used to generate random variants of the 847 GEICAM samples remaining after excluding those that could not be classified. The subtype classification reproducibility for each GEICAM sample was tested by generating 100,000 simulated samples using Monte Carlo simulations for each of the error models considered above, i.e. "best-," "average-" and "worst-case," for a total of 300,000 simulated samples per GEICAM sample. Based on the same sample justification analysis used for the archetypal samples, the calculated confidence interval width is ± 0.00087 for each simulation of 100,000 samples, which is an acceptable value.

### Error Effect on PAM50 Results for GEICAM Samples

After simulating 300,000 samples for each GEICAM sample based on the error models described above, subtype reproducibility for the individual samples was summarized for each tumor subtype (based on the original sample's subtype) using two statistics: (1) the total percentage of simulated samples that did (or did not) change subtypes with respect to their parent sample and (2) the proportion of simulated samples, corresponding to a single original sample, that were classified (or misclassified) identically to the parent sample. These data serve as an estimate of PAM50's misclassification rate based on intrinsic error within the assay when samples are not in proximity to the PAM50 centroids.

## Results and Discussion

### Archetypal Samples

All simulated samples derived from the archetypal samples were classified the same subtype as the parent sample, indicating that the PAM50 subtype classification for samples with characteristics close to the PAM50 centroids is highly reproducible and resilient to experimental errors in gene expression measurements. Although this positive result is encouraging for PAM50 classifications, it is not generalizable beyond the four archetypal samples.

### GEICAM 9906 Samples

Tables 1, 2, 3 present the results for the classification of all the samples produced by Monte Carlo simulation using the "best-" (Table 1), "average-" (Table 2) and "worst-case" (Table 3) scenarios for the error models described above. These scenarios correspond to using the lower values of the 95% confidence interval for the predicted error-model line, the actual predicted line and the upper values of the 95% confidence interval, respectively. The upper values of the 95% confidence interval are representative of the "worst-case" scenario because a greater amount of error is introduced into the simulation.

**Table 1:** Original GEICAM Sample Subtype vs Simulated Sample Subtype (as Percentage) - Best-Case Scenario

Original Subtype (simulated samples)	Classified as Subtype (%)				
	**Luminal A**	**Luminal B**	**HER2-enriched**	**Basal-like**	**Normal-like**

**Luminal A **(303000)	**96.12**	1.67	1.55	0	0.66

**Luminal B **(270000)	1.93	**97.27**	0.80	0	0

**HER2-enriched **(189000)	3.80	2.99	**92.25**	0.24	0.73

**Basal-like **(85000)	0	0	1.14	**98.86**	0

**Table 2:** Original GEICAM Sample Subtype vs. Simulated Sample Subtype (as Percentage) - Average-Case Scenario

Original Subtype (simulated samples)	Classified as Subtype (%)				
	**Luminal A**	**Luminal B**	**HER2-enriched**	**Basal-like**	**Normal-like**

**Luminal A **(303000)	**95.46**	1.98	1.74	0	0.81

**Luminal B **(270000)	2.34	**96.63**	1.03	0	0

**HER2-enriched **(189000)	4.69	3.76	**90.07**	0.45	1.03

**Basal-like **(85000)	0	0	1.51	**98.47**	0.02

**Table 3:** Original GEICAM Sample Subtype vs. Simulated Sample Subtype (as Percentage) - Worst-Case Scenario

Original Subtype (simulated samples)	Classified as Subtype (%)				
	**Luminal A**	**Luminal B**	**HER2-enriched**	**Basal-like**	**Normal-like**

**Luminal A **(303000)	**94.81**	2.29	1.93	0	0.97

**Luminal B **(270000)	2.75	**95.96**	1.29	0	0

**HER2-enriched **(189000)	5.65	4.45	**87.90**	0.67	1.33

**Basal-like **(85000)	0	0.01	1.98	**97.97**	0.04

Results for the GEICAM sample simulations suggest that all subtype classes are highly reproducible. When considering the three different error models, the most reproducible subtype among all GEICAM samples was always the Basal-like for which 98.47% (average-case) of the simulated samples did not change classification, followed by Luminal B (96.63%, average-case), Luminal A (95.46%, average-case) and HER2-enriched (90.07%, average-case). The differences in reproducibility between "best-" and "worst-case" ("best-case" percentages minus "worst-case" percentages) are 0.89%, 1.31%, 1.31% and 4.35% for Basal-like, Luminal B, Luminal A and HER2-enriched, respectively. Therefore, the selection of the error model is not a determining factor in assessing the robustness of the classification due to experimental errors in gene expression measurement.

**Figure 3:**
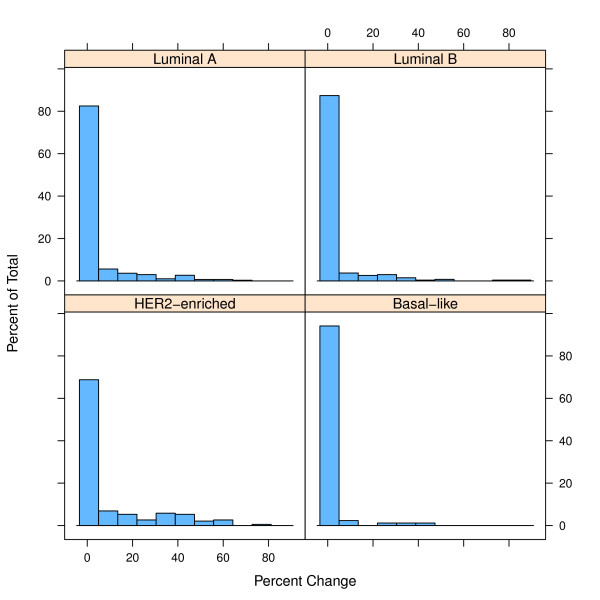
**Distribution of subtype reproducibility for replicas of individual GEICAM samples**. Each of the four histograms shows on the y-axis the percentage of the 1,000 replicas for which there are an x number of replicas that changed classification.

**Figure 4:**
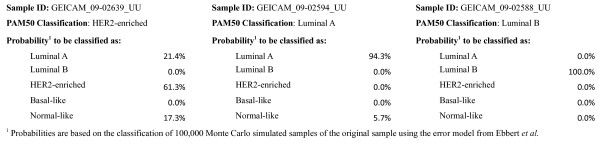
**Prototype of scorecard to report uncertainty in PAM50 classification due to intrinsic experimental errors in measuring gene expression factors using the example samples GEICAM_09-02639_UU, GEICAM_09-02594_UU and GEICAM_09-02588_UU**.

Figure 3 presents the histograms depicting the percentage of simulated samples, corresponding to a single parent sample, that change. Based on the "average-case" simulations and analyzing the classification of the 100,000 simulated samples generated for each sample we found that 80% (68 of 85) of Basal-like samples never change subtype during the Monte Carlo simulation, 69% (186 of 270) of Luminal B samples never change, 59% (178 of 303) of Luminal A samples never change and 22% (42 of 189) of HER2-enriched samples never change subtype. These values correspond approximately to the first bucket in the histogram. While the histograms confirm the results from Tables 1, 2, 3 on the general robustness of the PAM50 classification, they also reveal that there are a non-negligible number of samples for which a large number of simulated samples change classification. For instance the most variable sample for Basal-like, Luminal B, Luminal A and HER2-enriched changed in 42%, 86%, 67% and 75% of the simulated samples, respectively. As an example, the sample named "GEICAM_09-02639_UU" was originally classified as HER2-enriched, but 38.7% of its simulated samples were classified as something else. Specifically, the simulated samples were classified as HER2-enriched 61.3% of the time, Luminal A 21.4% of the time and Normal-like 17.3% of the time. These percentages could be translated into probabilities that can be reported to clinicians using a scorecard like the one depicted in Figure 4.

## Conclusions

We have developed a method based on Monte Carlo simulations and limited experimental measurements to estimate the effect of the intrinsic experimental errors in the measured factors contributing to MVAs. While the specifics of the error distribution functions given in Additional file 1 are not universal functions and they have to be recalculated for each lab and/or experimental setting, the method proposed here is generalizable and can be adapted to any MVA.

Using the proposed method based on Monte Carlo simulations and the error model described here, we have presented data that suggests PAM50's subtype classifications are highly reproducible on a large, independent sample set from the GEICAM 9906 clinical trial. We also show that there are a non-negligible number of samples for which a significant number of the Monte Carlo simulated samples classify differently than the parent sample, indicating that the classification of the original sample may not be reliable. To address this problem we suggest a new score card that can inform clinicians on the probability that a particular sample could be classified as a different tumor subtype.

## Competing interests

Dr. Philip Bernard is an inventor of the PAM50 breast test and is a co-founder of Bioclassifier LLC, which has licensing rights to the PAM50 signature.

## Authors' contributions

ME participated in the study design, performed all computational and statistical analyses, and drafted the manuscript. RB performed laboratory experiments for the repeated measures experiment. KB participated in the study design and edited the manuscript. IS performed laboratory experiments for the GEICAM series. MM designed and carried out GEICAM 9906 Clinical Trial. EC designed and carried out GEICAM 9906 Clinical Trial. RC designed and carried out GEICAM 9906 Clinical Trial. PB participated in the study design and coordination. JF participated in the study design and coordination, and edited the manuscript. All authors read and approved the final manuscript.

## Supplementary Material

Additional file 1 **Error Distributions**. Prior to performing Monte Carlo simulations to generate simulated samples, we needed to identify the distribution type that best models the repeated measures (error) data for each gene. Since twelve data points for each gene were not sufficient to identify a distribution type, data from each gene within each of the four archetypal samples were median-centered and combined, giving forty-seven data points per gene, since one of the archetypal Luminal A samples failed quality control. As shown in each of the fifty plots, the repeated measures data is most closely related to a Gaussian distribution, given the symmetry of most genes as well as the data clustering around a single mean value. Click here for file
