# Supplementary material for: Characterization of uncertainty in the classification of multivariate assays: application to PAM50 centroid-based genomic predictors for breast cancer treatment plans
Source: J Clin Bioinforma. 2011 Dec 23;1:37. doi: 10.1186/2043-9113-1-37 (PMC3275466; doi:10.1186/2043-9113-1-37)
Supplement: Additional file 1 — Error Distributions. Prior to performing Monte Carlo simulations to generate simulated samples, we needed to identify the distribution type that best models the repeated measures (error) data for each gene. Since twelve data points for each gene were not sufficient to identify a distribution type, data from each gene within each of the four archetypal samples were median-centered and combined, giving forty-seven data points per gene, since one of the archetypal Luminal A samples failed quality control. As shown in each of the fifty plots, the repeated measures data is most closely related to a Gaussian distribution, given the symmetry of most genes as well as the data clustering around a single mean value. [file 2043-9113-1-37-S1.PDF]

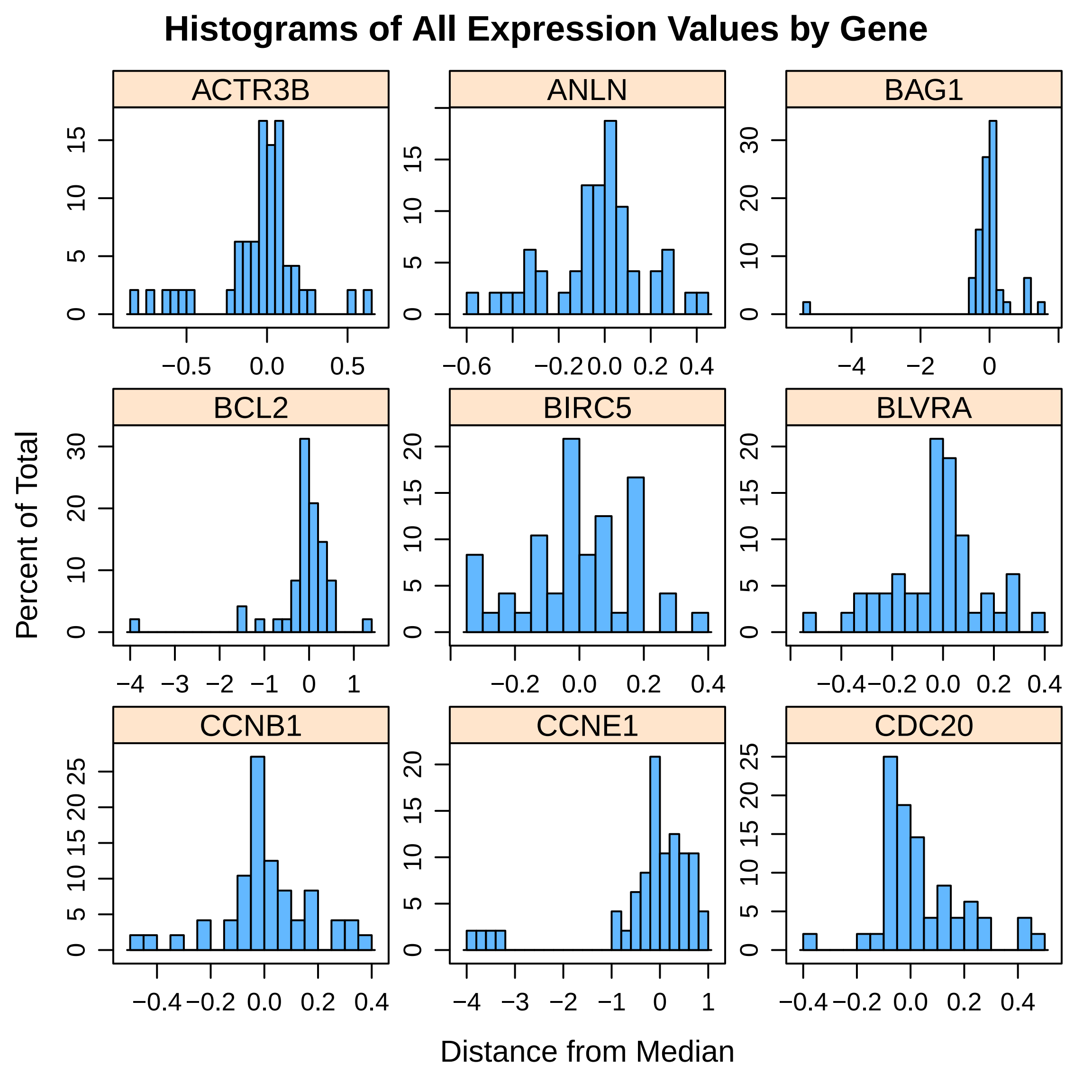

Histograms of All Expression Values by Gene
ACTR3B
ANLN
BAG1
BCL2
BIRC5
BLVRA
CCNB1
CCNE1
CDC20
Percent of Total
Distance from Median

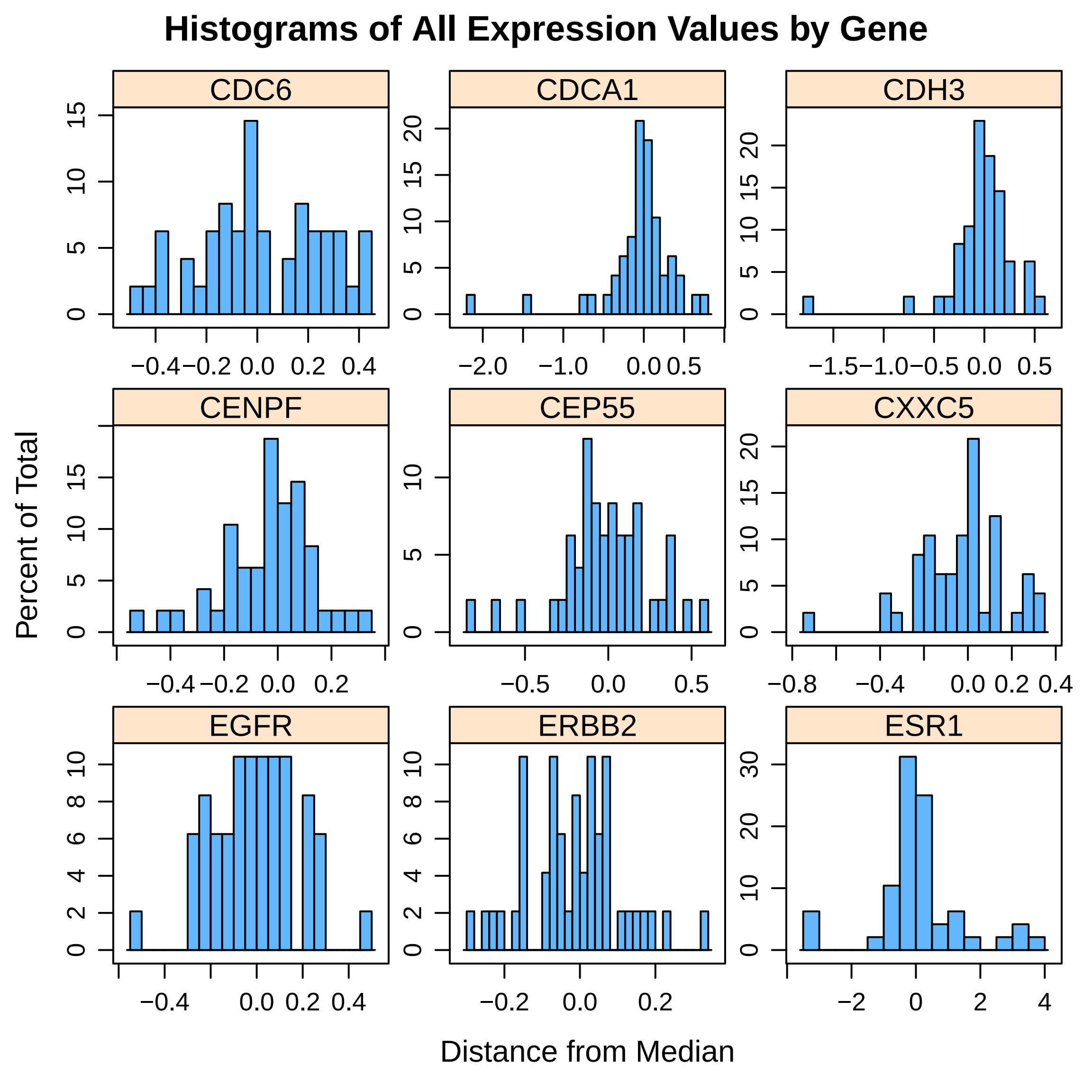

Histograms of All Expression Values by Gene
CDC6
CDCA1
CDH3
CENPF
CEP55
CXXC5
EGFR
ERBB2
ESR1
Percent of Total
Distance from Median

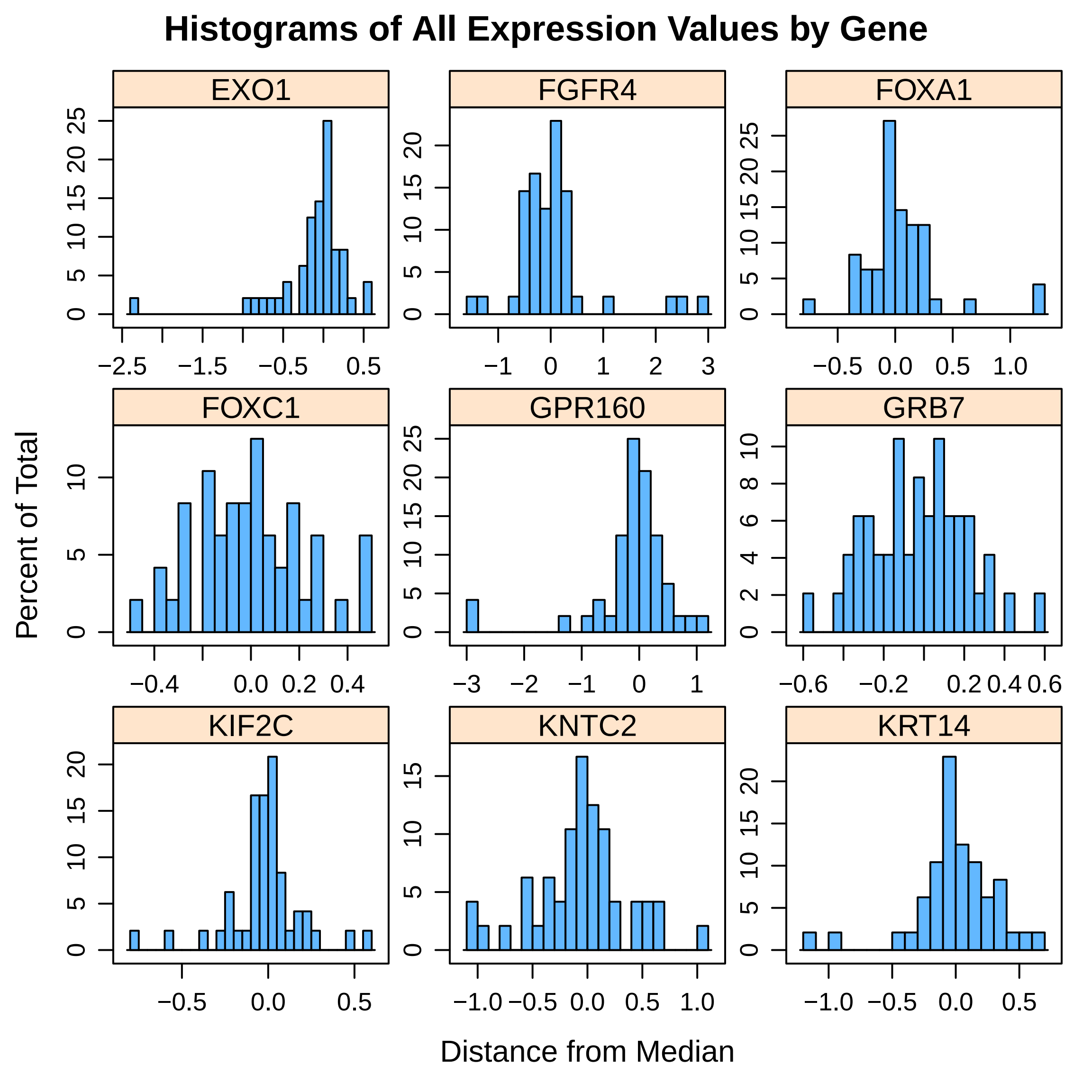

Histograms of All Expression Values by Gene
EXO1
FGFR4
FOXA1
FOXC1
GPR160
GRB7
KIF2C
KNTC2
KRT14
Percent of Total
Distance from Median

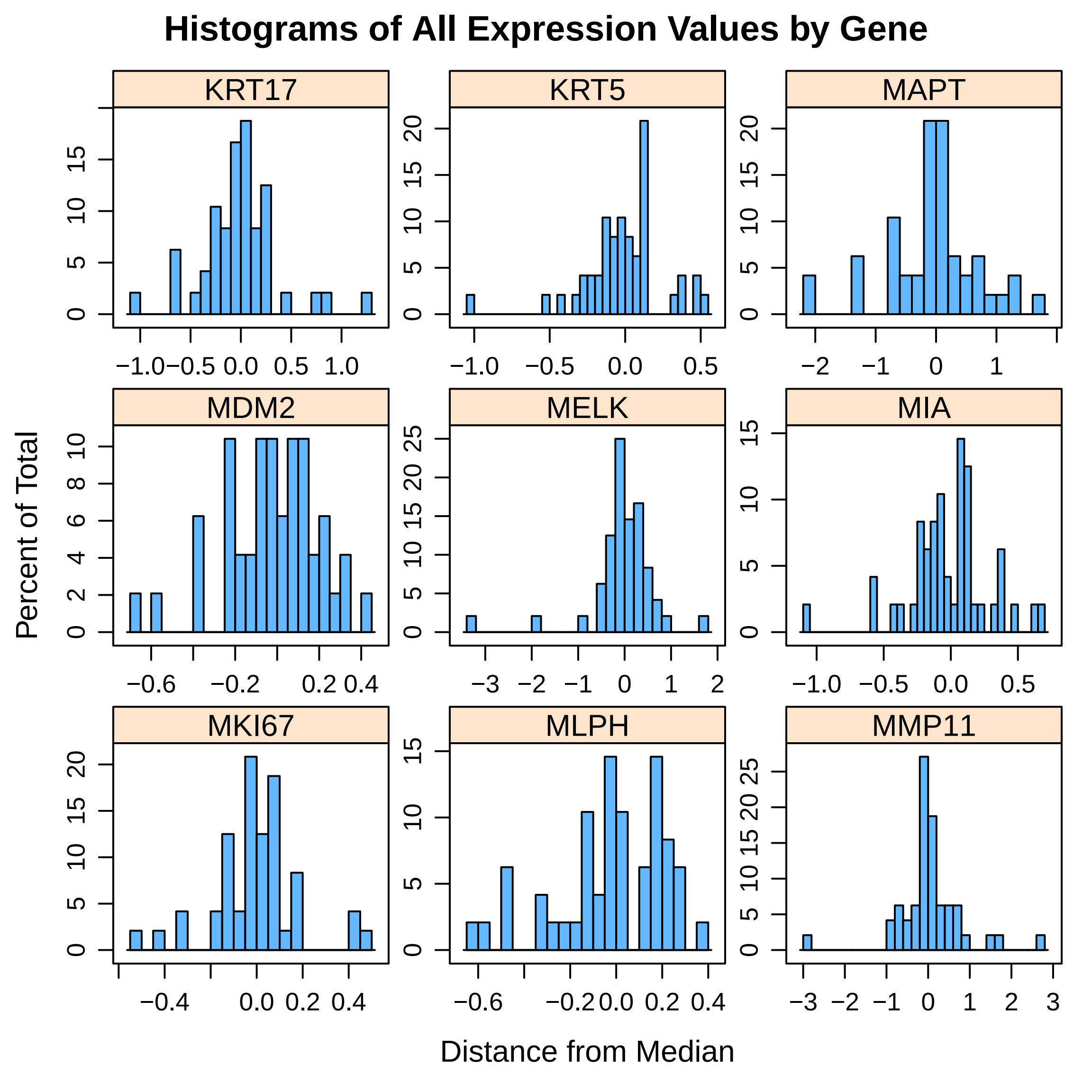

Histograms of All Expression Values by Gene
KRT17
KRT5
MAPT
MDM2
MELK
MIA
MKI67
MLPH
MMP11
Percent of Total
Distance from Median

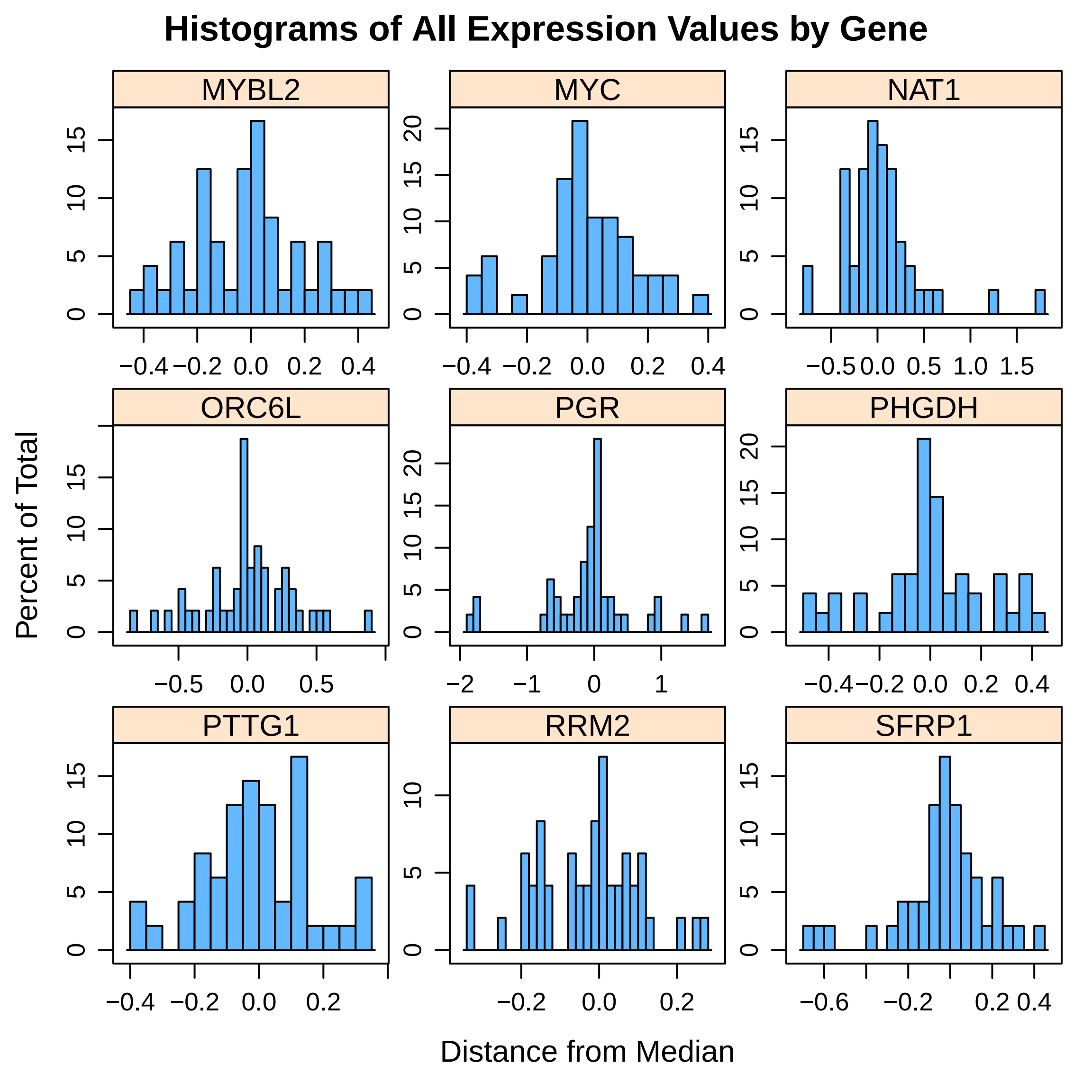

Histograms of All Expression Values by Gene
MYBL2
MYC
NAT1
ORC6L
PGR
PHGDH
PTTG1
RRM2
SFRP1
Percent of Total
Distance from Median

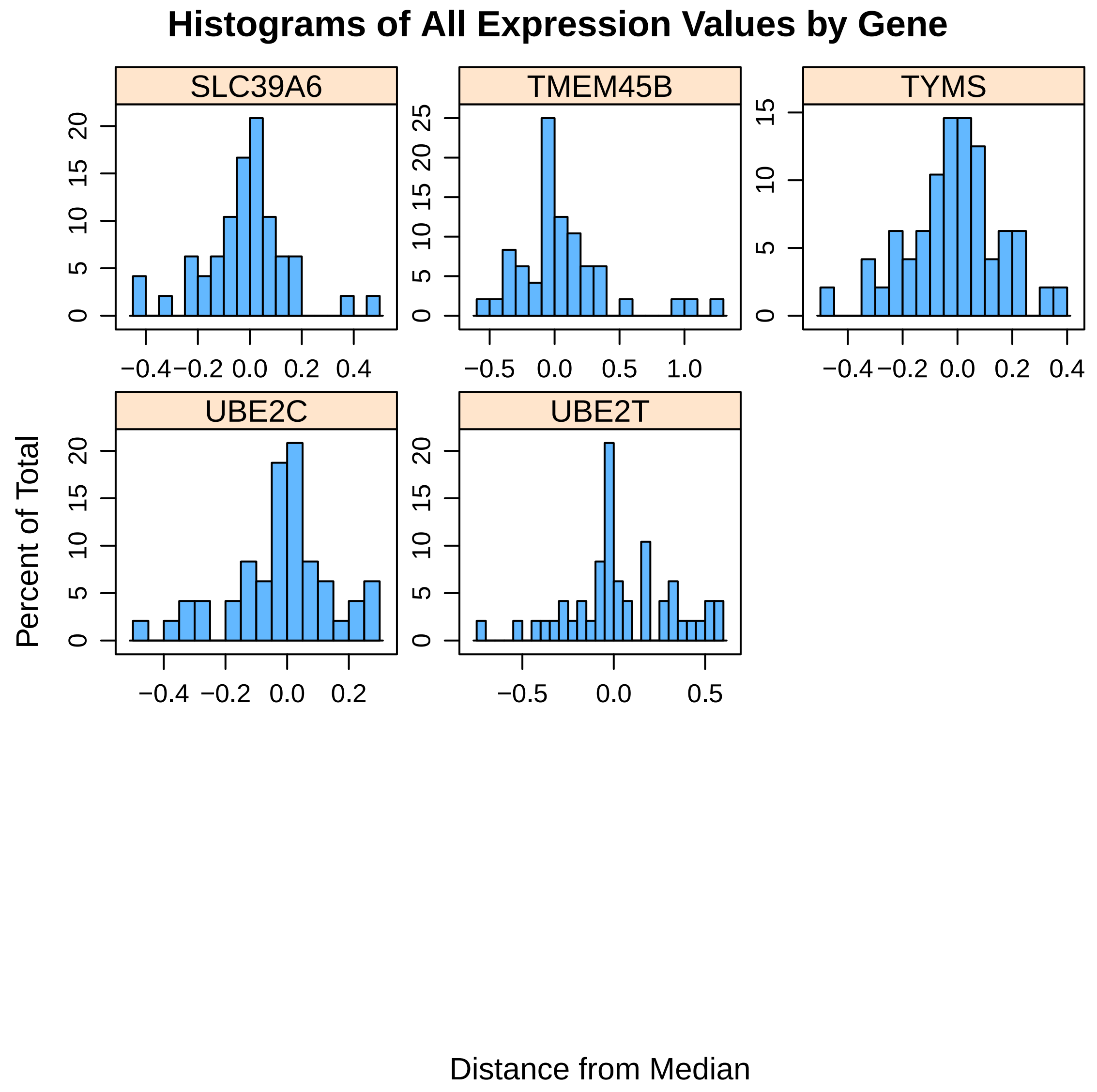

Histograms of All Expression Values by Gene
SLC39A6
TMEM45B
TYMS
UBE2C
UBE2T
Percent of Total
Distance from Median
